# Supplementary material for: Analysis of PPARγ Signaling Activity in Psoriasis
Source: Int J Mol Sci. 2021 Aug 10;22(16):8603. doi: 10.3390/ijms22168603 (PMC8395241; doi:10.3390/ijms22168603)
Supplement: Supplementary file 1 [file ijms-22-08603-s001.zip › Supplemental materials_Analysis of PPARg signaling activity in psoriasis/Pathway models/Models images and html files/Model of PPARG signaling in psoriasis/11610.html]

TRAF2


# Protein TRAF2

|  |  |
| --- | --- |
| URN | urn:agi-llid:7186 |
| Total Entities | 0 |
| Connectivity | 1802 |
| Name | TRAF2 |
| Description | TNF receptor associated factor 2 |
| Notes | The protein encoded by this gene is a member of the TNF receptor associated factor (TRAF) protein family. TRAF proteins associate with, and mediate the signal transduction from members of the TNF receptor superfamily. This protein directly interacts with TNF receptors, and forms a heterodimeric complex with TRAF1. This protein is required for TNF-alpha-mediated activation of MAPK8/JNK and NF-kappaB. The protein complex formed by this protein and TRAF1 interacts with the inhibitor-of-apoptosis proteins (IAPs), and functions as a mediator of the anti-apoptotic signals from TNF receptors. The interaction of this protein with TRADD, a TNF receptor associated apoptotic signal transducer, ensures the recruitment of IAPs for the direct inhibition of caspase activation. BIRC2/c-IAP1, an apoptosis inhibitor possessing ubiquitin ligase activity, can unbiquitinate and induce the degradation of this protein, and thus potentiate TNF-induced apoptosis. Multiple alternatively spliced transcript variants have been found for this gene, but the biological validity of only one transcript has been determined. [provided by RefSeq, Jul 2008] |
| Primary Cell Localization | Cytoplasm |

---

|  |  |
| --- | --- |
| Pathway | Osteoarthritis |
|  | Skin Aging |
|  | MTHFR Activity Regulation |
|  | TLR4 Signaling in Leukocytes |
|  | T-Cell Independent B-Cell Activation |
|  | Immunoglobulin Class-Switch Recombination Activation |
|  | T-Cell Maturation (Hypothesis) |
|  | Peripheral T-Cell Tolerance |
|  | Necrosis |
|  | Leukotriene Effect on Vascular Endothelial Cell Response |
|  | Eosinophil Survival by Cytokine Signaling |
|  | Mast-Cell Activation without Degranulation through TNFSF8 Signaling |
|  | Mast-Cell Activation without Degranulation |
|  | Macrophage M1 Lineage |
|  | Neutrophil Recruitment and Priming |
|  | Negative Acute Phase Proteins Synthesis |
|  | Positive Acute Phase Proteins Synthesis |
|  | Vascular Endothelial Cell Activation by Cytokines |
|  | Adipokines Production by Adipocyte |
|  | Lipolysis Regulation in Adipocyte |
|  | GFs/TNF -> Ion Channels |
|  | Circadian Clock Genes in Suprachiasmatic Nuclei Neurons |
|  | Apoptosis |
|  | SCF/FBXO7 Complex |
|  | Dilated Cardiomyopathy |
|  | Cardiomyocyte Hypertrophy |
|  | Hypertrophic Cardiomyopathy |
|  | Myocardial Infarction |
|  | Inflammatory Reaction in Acne Vulgaris |
|  | Th0-Cell Aberrant Activation in Systemic Lupus Erythematosus |
|  | B-Cell Activation in Systemic Lupus Erythematosus |
|  | Toll-like Receptors in beta-Cell |
|  | Podocyte Dysfunction in Diabetic Nephropathy |
|  | Adipose Tissue Inflammation Provokes Insulin Resistance |
|  | Oxidation of Fatty Acid in non-Alcoholic Fatty Liver Disease |
|  | beta-Cell Destruction through Cytokines in Diabetes Mellitus |
|  | Nitric Oxide Effects on beta-Cell |
|  | Thymic Follicular Hyperplasia |
|  | Adipokines Production by Adipocyte Impaired in Obesity |
|  | Crohn's Disease |
|  | Macrophage Dysfunction |
|  | T-Cell Cytotoxic Response in Ulcerative Colitis |
|  | Diffuse Large-B-Cell Lymphoma |
|  | Follicular Lymphoma |
|  | Hodgkin Lymphoma |
|  | Mantle Cell Lymphoma |
|  | mTOR/NF-kB/BCR Signaling Disregulation in Mantle Cell Lymphoma |
|  | Splenic Marginal Zone Lymphoma |
|  | Proteins Involved in Marginal Zone Lymphoma |
|  | TNFR/NF-kB Classical Pathway in Multiple Myeloma Plasma Cells |
|  | Osteoclast Activation in Multiple Myeloma |
|  | Multiple Myeloma |
|  | TNFR/NF-kB Alternative Pathway in Multiple Myeloma Plasma Cells |
|  | SERPINA1 Associated Liver Damage |
|  | Eosinophil Survival in Asthma |
|  | Goblet-Cell Related Mucus Secretion in Asthma |
|  | Club-Cell Role in Asthma |
|  | B-Cell Mediated IgE Production in Asthma |
|  | CFTR Facilitates Oxidative Stress in Airway Epithelium in CF |
|  | Airway Epithelial Cell Dysfunction |
|  | Osteoclast Activation in Gout |
|  | Neutrophil Recruitment in Sinovium in Gout |
|  | Hyper IgM Syndrome |
|  | TNF and IL1B Induce Metalloproteinase Synthesis in Osteoarthritis |
|  | Apoptosis of Chondrocyte |
|  | Dendritic Cell Dysfunction in Psoriatic Arthritis |
|  | Osteoclast Activation in Psoriatic Arthritis |
|  | Synovial Fibroblast Activation in Psoriatic Arthritis |
|  | Osteoclast Activation in Rheumatoid Arthritis |
|  | Synovial Fibroblast Activation by Citokines in Rheumatoid Arthritis |
|  | Apoptosis Block in Synovial Fibroblast |
|  | Skin Fibrosis |
|  | Dentin Formation Disruption |
|  | Apoptosis Block in Infected Cells |
|  | Diabetes Induced Periodontitis |
|  | IL1 Receptor Antagonist (IL1RN) Deficiency (DIRA) |
|  | TNF Receptor Associated Periodic Syndrome |
|  | ER Stress in Huntington Disease |
|  | Osteoclast Activation in Postmenopause |
|  | Proteins Involved in Glioblastoma |
|  | Growth Factor Signaling in Neuroblastoma |
|  | Neuroblastoma |
|  | Crystallin alpha B (CRYAB) Suppresses Multiple Sclerosis |
|  | Breast Cancer |
|  | Subepithelial Fibroblasts in Middle Ear Cholesteatoma |
|  | HPV E6 and E7 Induced Disruption of TNFR/NF-kB Signaling in Keratinocytes |
|  | ER Stress in Macular Degeneration |
|  | OPTN Effects on NF-kB Signaling in Glaucoma |
|  | MYOC Associated Glaucoma |
|  | Neurotrophic Factor Deprivation in Retinal Ganglion Cell Death |
|  | Alveolar Macrophages Dysfunction in COPD |
|  | Mucin Hyperproduction in Goblet and Mucous Cells |
|  | Endothelial Cell Dysfunction in Glomerulonephritis |
|  | Podocyte Dysfunction in Glomerulonephritis |
|  | Interstitial Fibroblasts in Pyelonephritis |
|  | Apoptosis of Renal Tubule Epithelial Cell in Pyelonephritis |
|  | Endothelial Cell Dysfunction in Pyelonephritis |
|  | NFKB Canonical Signaling Activation in Cancer |
|  | NFKB Non-Canonical Signaling Activation in Cancer |
|  | STAT3 and NFkB Activate Inflammation-Induced Tumorigenesis |
|  | NFKB Signaling Activation by Blocking of Tumor Suppressors |
|  | Apoptosis Block by Death Receptor Signaling in Cancer |
|  | Apoptosis Evasion in Cancer: Overview |
|  | Cancer Cells Inhibit Adipocyte Differentiation |
|  | Skeletal Muscle Wasting in Cancer Cachexia |
|  | IDO1 in Cancer Immune Escape |
|  | Effector T-cell Inactivation in Cancer Immune Escape |
|  | CD40 -> Expression Targets in Lymph Node |
|  | NGF -> SMAD3/NF-kB Expression Targets |
|  | NGF -> AP-1/TP53/MYC Expression Targets |
|  | NGF -> FOXO/MYCN/ELK-SRF Expression Targets |
|  | BDNF Expression Targets |
|  | NTF3 Expression Targets |
|  | NTF4 Expression Targets |
|  | NGF -> CREB/CEBPB/MEF2A Expression Targets |
|  | TGFBR -> MEF/MYOD/MYOG Signaling |
|  | TNF-alpha/TNFRSF1A Signaling |
|  | JNK/MAPK Signaling |
|  | P38 MAPK/MAPK14 Signaling |
|  | NF-kB non-Canonical Signaling |
|  | TNF-alpha/TNFRSF1B Signaling |
|  | NF-kB Canonical Signaling |
|  | FASLG Expression Targets |
|  | TNF -> NF-kB Expression Targets |
|  | TNFSF10 Expression Targets |
|  | LTA Expression Targets |
|  | TNFSF14 Expression Targets |
|  | TNF -> AP-1 Expression Targets |
|  | CD72 -> AP-1 Expression Targets |
|  | CD40LG -> NF-kB/ELK/SRF -> CREB/NFATC Expression Targets |
|  | TNFSF13 Expression Targets |
|  | TNFSF13B Expression Targets |
|  | TNF -> ELK-SRF Expression Targets |
|  | TNF -> CREB Expression Targets |
|  | TNF -> TP53/ATF Expression Targets |
|  | EDA Expression Targets |
|  | EctodysplasinR -> NF-kB Signaling |
|  | EctodysplasinR -> AP-1 Signaling |
|  | TNFR -> CREB/ELK-SRF Signaling |
|  | TNFR -> AP-1/ATF/TP53 Signaling |
|  | NGFR -> AP-1/CEBPB/CREB/ELK/SRF/TP53 Signaling |
|  | TNFRSF5/6 -> RB1/E2F Signaling |
|  | NGFR -> NF-kB Signaling |
|  | EctodysplasinR -> LEF1 Signaling |
|  | TNFR -> NF-kB Signaling |
|  | NGFR -> MEF Signaling |
|  | TNFRSF6 -> DDIT3 Signaling |
|  | TNFRSF6 -> HSF1 Signaling |
|  | TNFRSF1A -> AP-1/ATF/TP53 Signaling |
|  | TNFRSF1A -> CREB/ELK-SRF Signaling |
|  | ER Stress (Unfolded Protein Response) |
|  | Model of PPARG signaling in psoriasis |
|  | 1\_TNF and IL1B provoke ECM degradation in osteoarthritis |
|  | 1\_Osteoclast-mediated bone resorption in postmenopausal women |
|  | 2\_Chondrocyte death in osteoarthritis |
|  | 2\_The role of endothelial cells in pyelonephritis |
|  | 3\_The role of interstitial fibroblasts in pyelonephritis |
|  | 2\_Podocyte dysfunction in glomerulonephritis |
|  | 3\_Role of neurotrophic factor deprivation in retinal ganglion cell death |
|  | 2\_MYOC-associated glaucoma |
|  | 3\_Endoplasmic reticulum stress in age-related macular degeneration |
|  | 2\_Pathogens stimulate mucins expression in the middle ear |
|  | 2\_Cardiomyocyte hypertrophy |
|  | 2\_Cigarette smoke and inflammation cause alveolar epithelial cell death in COPD |
|  | 3\_Role of alveolar macrophages in pulmonary tissue destruction in COPD |
|  | 3\_2\_Regulation of airway surface liquid (ASL) thickness |
|  | 3\_1\_Mucus accumulation in asthma |
|  | 2\_2\_Eosinophilia and neutrophilia in asthma: eosinophil apoptosis |
|  | 4\_Mucin hyperproduction in goblet and mucous cells in COPD |
|  | 1\_Defects in response to pathogens in the gut promotes inflammation in Crohn's disease |
|  | 3\_Autophagy dysfunction in Crohn's disease |
|  | 2\_Lipotoxicity in NAFLD |
|  | 6\_p12I Viral protein promotes T-cell activation in HTLV-1 infection |
|  | 5\_Human papilloma virus E6 and E7 proteins promote epithelial cell proliferation |
|  | 2\_HIV - induced depletion of CD4+ T-cells |
|  | 4\_Inflammatory mediators inhibit GATA signaling in erythropoiesis |
|  | 4\_2\_Insulin resistance: inflammation-related insulin resistance |
|  | 2\_SERPINA1-associated liver damage |
|  | 1\_Antigen-presenting cells promote islet dysfunction and immune system activation in T1D |
|  | 2\_Neuron and Schwann cell death |

---

|  |  |
| --- | --- |
| Group | Genes with Mutations Associated with Multiple Myeloma |

---

|  |  |
| --- | --- |
| MedScan ID | 7186 |

---

|  |  |
| --- | --- |
| LocusLink ID | 7186 |
|  | 22030 |
|  | 311786 |
|  | 98924 |

---

|  |  |
| --- | --- |
| Alias | AI325259 |
|  | tumor necrosis factor type 2 receptor associated protein 3 |
|  | TRAF2 |
|  | LOC203234 |
|  | OTTHUMP00000022625 |
|  | OTTHUMP00000064745 |
|  | TNF receptor-associated factor II |
|  | E3 ubiquitin-protein ligase TRAF2 |
|  | TNF receptor-associated factor 2 |
|  | OTTMUSP00000013079 |
|  | RING-type E3 ubiquitin transferase TRAF2 |
|  | TRA2\_HUMAN |
|  | OTTMUSP00000013078 |
|  | Tumor necrosis factor receptor associated factor 2 |
|  | RNF117 |
|  | Tumor necrosis factor type 2 receptor associated protein III |
|  | MGC:45012 |
|  | RP23-464C2.1 |
|  | TRAP3 |
|  | TRA2 |
|  | TRAP |
|  | tumour necrosis factor type 2 receptor associated protein III |
|  | tumour necrosis factor receptor associated factor 2 |
|  | tumour necrosis factor type 2 receptor associated protein 3 |
|  | tumor necrosis factor type 2 receptor-associated protein 3 |
|  | Traf2\_predicted |
|  | Tumor necrosis factor type 2 receptor associated protein 3 |
|  | Tumor necrosis factor type 2 receptor associated protein III |
|  | Tnf receptor-associated factor 2 (predicted) |

---

|  |  |
| --- | --- |
| EC Number | 2.3.2.27 |
|  | 6.3.2.- |

---

|  |  |
| --- | --- |
| GO ID | 0005174 |
|  | 0019899 |
|  | 0031435 |
|  | 0019901 |
|  | 0019903 |
|  | 0044877 |
|  | 0046625 |
|  | 0031996 |
|  | 0005164 |
|  | 0031625 |
|  | 0004842 |
|  | 0008270 |
|  | 0007249 |
|  | 0007250 |
|  | 0006919 |
|  | 0034622 |
|  | 0071732 |
|  | 0071550 |
|  | 0070059 |
|  | 1902042 |
|  | 0034351 |
|  | 1901215 |
|  | 0051091 |
|  | 1903721 |
|  | 0043507 |
|  | 0051092 |
|  | 0002726 |
|  | 2001238 |
|  | 0032743 |
|  | 0090073 |
|  | 1903265 |
|  | 0097300 |
|  | 0070534 |
|  | 0051865 |
|  | 0030163 |
|  | 0016579 |
|  | 0051291 |
|  | 0070207 |
|  | 0065003 |
|  | 0042981 |
|  | 1902041 |
|  | 0051023 |
|  | 0010803 |
|  | 0034976 |
|  | 0007165 |
|  | 0033209 |
|  | 0035631 |
|  | 1990604 |
|  | 0097057 |
|  | 0005938 |
|  | 0009898 |
|  | 0005829 |
|  | 0045121 |
|  | 0005654 |
|  | 0032991 |
|  | 0000151 |
|  | 0012506 |
|  | 0008289 |
|  | 0046872 |
|  | 0016740 |
|  | 0006915 |
|  | 0043066 |
|  | 0043123 |
|  | 0046328 |
|  | 0005737 |
|  | 1990597 |
|  | 0016874 |
|  | 0032403 |
|  | 0004871 |
|  | 0097296 |
|  | 0043623 |
|  | 0050870 |
|  | 0006461 |
|  | 0010939 |
|  | 0008766 |
|  | 0043773 |
|  | 0043774 |
|  | 0070739 |
|  | 0070735 |
|  | 0070737 |
|  | 0070736 |
|  | 0005102 |
|  | 0018169 |
|  | 0070740 |
|  | 0070738 |
|  | 0043234 |
|  | 0045087 |
|  | 2001239 |
|  | 0097190 |
|  | 0008633 |
|  | 0008624 |
|  | 0006916 |
|  | 0004872 |
|  | 0008152 |
|  | 0005515 |

---

|  |  |
| --- | --- |
| KEGG ID | hsa:7186 |
|  | mmu:22030 |
|  | rno:311786 |

---

|  |  |
| --- | --- |
| MedLine Reference | 10349636 |
|  | 10514016 |
|  | 10518213 |
|  | 10702415 |
|  | 11042159 |
|  | 11076861 |
|  | 11217851 |
|  | 11275257 |
|  | 11479302 |
|  | 11907088 |
|  | 11909853 |
|  | 12021180 |
|  | 12133933 |
|  | 12218092 |
|  | 12354380 |
|  | 12370254 |
|  | 12411493 |
|  | 12466851 |
|  | 12477932 |
|  | 12529173 |
|  | 12711536 |
|  | 12842894 |
|  | 12897154 |
|  | 12958312 |
|  | 12960157 |
|  | 14634111 |
|  | 14701813 |
|  | 15199146 |
|  | 15485859 |
|  | 15539150 |
|  | 15545599 |
|  | 15590671 |
|  | 15782199 |
|  | 15824857 |
|  | 16141072 |
|  | 16141073 |
|  | 16147974 |
|  | 16148124 |
|  | 16299380 |
|  | 16446354 |
|  | 16446357 |
|  | 16622006 |
|  | 16953841 |
|  | 8069916 |
|  | 8565075 |
|  | 9104814 |
|  | 9177772 |
|  | 9390694 |
|  | 9461607 |
|  | 9488716 |
|  | 9507120 |
|  | 9692890 |
|  | 9916988 |
|  | 10025951 |
|  | 10037686 |
|  | 10339433 |
|  | 10346818 |
|  | 10411888 |
|  | 10477597 |
|  | 10490605 |
|  | 10514511 |
|  | 10521462 |
|  | 10523862 |
|  | 10581243 |
|  | 10617615 |
|  | 10650002 |
|  | 10753917 |
|  | 10764746 |
|  | 10809768 |
|  | 10880535 |
|  | 10892748 |
|  | 10903733 |
|  | 10990461 |
|  | 11035039 |
|  | 11064452 |
|  | 11112773 |
|  | 11278268 |
|  | 11278723 |
|  | 11279055 |
|  | 11340079 |
|  | 11359906 |
|  | 11374864 |
|  | 11435475 |
|  | 11466612 |
|  | 11728344 |
|  | 11777919 |
|  | 11798190 |
|  | 11804591 |
|  | 11805080 |
|  | 11882293 |
|  | 11907583 |
|  | 11932422 |
|  | 11971184 |
|  | 12136106 |
|  | 12169272 |
|  | 12411322 |
|  | 12547194 |
|  | 12571250 |
|  | 12591926 |
|  | 12843613 |
|  | 12887920 |
|  | 12917691 |
|  | 14557256 |
|  | 14585990 |
|  | 14633987 |
|  | 14702039 |
|  | 14741690 |
|  | 14743216 |
|  | 15125833 |
|  | 15208311 |
|  | 15265700 |
|  | 15308666 |
|  | 15310755 |
|  | 15383523 |
|  | 15670977 |
|  | 15708970 |
|  | 15743837 |
|  | 15944293 |
|  | 16009713 |
|  | 16189514 |
|  | 16227629 |
|  | 16260598 |
|  | 16282325 |
|  | 16304992 |
|  | 16311516 |
|  | 16636664 |
|  | 16713569 |
|  | 16891304 |
|  | 16936264 |
|  | 7639698 |
|  | 8612133 |
|  | 8627180 |
|  | 8643514 |
|  | 8692885 |
|  | 8702708 |
|  | 8710854 |
|  | 8875942 |
|  | 8943059 |
|  | 8985011 |
|  | 8999898 |
|  | 9020361 |
|  | 9153189 |
|  | 9162022 |
|  | 9168896 |
|  | 9208847 |
|  | 9353251 |
|  | 9373149 |
|  | 9384571 |
|  | 9418902 |
|  | 9464265 |
|  | 9500555 |
|  | 9582383 |
|  | 9607925 |
|  | 9705938 |
|  | 9712898 |
|  | 9733516 |
|  | 9774460 |
|  | 9774977 |
|  | 9827693 |
|  | 9852070 |
|  | 10206649 |
|  | 10463949 |
|  | 11384837 |
|  | 11607847 |
|  | 11784851 |
|  | 15489334 |
|  | 16153868 |
|  | 16214042 |
|  | 17379600 |
|  | 17389591 |
|  | 8125298 |
|  | 9718306 |
|  | 10748240 |
|  | 17015619 |
|  | 17079781 |
|  | 17277132 |
|  | 17332487 |
|  | 17360936 |
|  | 17462996 |
|  | 17572386 |
|  | 15590916 |

---

|  |  |
| --- | --- |
| Organism | Homo sapiens {Organism urn:agi-taxid:9606} |
|  | Mus musculus {Organism urn:agi-taxid:10090} |
|  | Rattus norvegicus {Organism urn:agi-taxid:10116} |
|  | Homo sapiens |
|  | Mus musculus |
|  | Rattus norvegicus |

---

|  |  |
| --- | --- |
| Mouse chromosome position | 2 |
|  | 2 A3 |

---

|  |  |
| --- | --- |
| OMIM ID | 601895 |

---

|  |  |
| --- | --- |
| Rat chromosome position | 3p13 |

---

|  |  |
| --- | --- |
| Hugo ID | 12032 |
|  | HGNC:12032 |

---

|  |  |
| --- | --- |
| Human chromosome position | 9q34.3 |
|  | 9q34 |

---

|  |  |
| --- | --- |
| Swiss-Prot Accession | A0A024R8H5 |
|  | Q12933 |
|  | Q12933.2 |
|  | P39429 |
|  | Q8C6X9 |
|  | Q3U8L1 |
|  | P39429.1 |
|  | B5DFH7 |
|  | A8K107 |
|  | B4DPJ7 |
|  | Q7Z337 |
|  | Q96NT2 |
|  | A2AJA3 |
|  | O54896 |
|  | Q3TWR1 |
|  | Q8CAQ7 |
|  | TRAF2\_HUMAN |
|  | TRAF2\_MOUSE |
|  | A8K107\_HUMAN |
|  | B5DFH7\_RAT |
|  | Q3TWR1\_MOUSE |
|  | Q3U8L1\_MOUSE |
|  | Q5T5T6 |
|  | Q5T1L8 |
|  | Q53GH0 |
|  | Q5T1L7 |
|  | Q5T1L6 |
|  | Q5T1L5 |

---

|  |  |
| --- | --- |
| PIR ID | S56163 |
|  | I61512 |

---

|  |  |
| --- | --- |
| GenBank ID | NC\_000009 |
|  | XM\_011518976 |
|  | XP\_011517278 |
|  | XM\_011518977 |
|  | XP\_011517279 |
|  | NM\_021138 |
|  | NP\_066961 |
|  | XM\_011518974 |
|  | XP\_011517276 |
|  | AL355987 |
|  | AL449425 |
|  | AY623660 |
|  | AAT27320 |
|  | CH471090 |
|  | EAW88299 |
|  | EAW88300 |
|  | EAW88301 |
|  | EAW88302 |
|  | EAW88303 |
|  | EAW88304 |
|  | EAW88305 |
|  | KT585270 |
|  | AK054686 |
|  | BAB70792 |
|  | AK222961 |
|  | AK289722 |
|  | BAF82411 |
|  | AK294607 |
|  | BAG57794 |
|  | AK298370 |
|  | BAG60609 |
|  | BC032410 |
|  | AAH32410 |
|  | BC033810 |
|  | AAH33810 |
|  | BC043492 |
|  | AAH43492 |
|  | BC064662 |
|  | AAH64662 |
|  | BM785733 |
|  | BX538160 |
|  | CAD98040 |
|  | HM991672 |
|  | ADQ89802 |
|  | Q12933 |
|  | NC\_000068 |
|  | NM\_009422 |
|  | NP\_033448 |
|  | NM\_001290413 |
|  | NP\_001277342 |
|  | XM\_006497850 |
|  | XP\_006497913 |
|  | XM\_006497849 |
|  | XP\_006497912 |
|  | AH009199 |
|  | AAF59928 |
|  | AL732590 |
|  | CH466542 |
|  | EDL08257 |
|  | AF027570 |
|  | AAC53545 |
|  | AK038136 |
|  | BAC29937 |
|  | AK052934 |
|  | BAC35209 |
|  | AK151653 |
|  | BAE30582 |
|  | AK152174 |
|  | BAE31006 |
|  | AK159581 |
|  | BAE35201 |
|  | AK159585 |
|  | BAE35205 |
|  | AK184085 |
|  | AK184736 |
|  | AK190736 |
|  | AK199540 |
|  | BB841262 |
|  | BC003801 |
|  | AAH03801 |
|  | BC060625 |
|  | L35303 |
|  | AAC37662 |
|  | P39429 |
|  | NC\_005102 |
|  | XM\_008761588 |
|  | XP\_008759810 |
|  | XM\_006233596 |
|  | XP\_006233658 |
|  | XM\_006233594 |
|  | XP\_006233656 |
|  | NM\_001107815 |
|  | NP\_001101285 |
|  | AC\_000071 |
|  | AABR07051243 |
|  | AAHX01020458 |
|  | CH474001 |
|  | EDL93568 |
|  | BC169064 |
|  | AAI69064 |
|  | XM\_011518973 |
|  | XP\_011517275 |
|  | XM\_005266099 |
|  | XP\_005266156 |
|  | XM\_011518975 |
|  | XP\_011517277 |
|  | XM\_011518978 |
|  | XP\_011517280 |
|  | NC\_018920 |
|  | AMYH02021914 |
|  | DQ893616 |
|  | ABM84542 |
|  | DQ894764 |
|  | ABM85690 |
|  | EU831941 |
|  | ACE87382 |
|  | EU831961 |
|  | ACE87402 |
|  | EU832036 |
|  | ACE86698 |
|  | EU832054 |
|  | ACE86716 |
|  | AC\_000024 |
|  | AAHY01014738 |
|  | AAHY01014739 |
|  | AF233325 |
|  | AF233326 |
|  | AF233327 |
|  | AF233328 |
|  | AF233329 |
|  | AF233330 |
|  | AF233331 |
|  | AF233332 |
|  | AC\_000141 |
|  | ABBA01022731 |
|  | ABBA01022732 |
|  | XM\_006497847 |
|  | XP\_006497910 |
|  | XM\_006497848 |
|  | XP\_006497911 |
|  | XM\_005266098 |
|  | XP\_005266155 |
|  | XM\_005266101 |
|  | XP\_005266158 |
|  | XM\_005266100 |
|  | XP\_005266157 |
|  | XM\_006233595 |
|  | XP\_006233657 |
|  | AABR06021895 |
|  | CAI12701 |
|  | CAI12702 |
|  | CAI12703 |
|  | CAI12704 |
|  | CAI12705 |
|  | CAI15103 |
|  | CAI15104 |
|  | CAI15105 |
|  | CAI15106 |
|  | CAM25164 |
|  | CAM25165 |
|  | NT\_024000 |
|  | NW\_924573 |
|  | NW\_001839245 |
|  | AC\_000052 |
|  | CR611225 |
|  | CR624454 |
|  | NT\_039206 |
|  | NW\_001030686 |
|  | Q3TWR1 |
|  | Q3U8L1 |
|  | Q8C6X9 |
|  | Q8CAQ7 |
|  | NW\_047651 |
|  | NW\_001084810 |
|  | Q7Z337 |
|  | XM\_231032 |
|  | XP\_231032 |
|  | XM\_001077727 |
|  | XP\_001077727 |
|  | Q53GH0 |
|  | Q5T1L5 |
|  | Q5T1L6 |
|  | Q5T1L7 |
|  | Q5T1L8 |
|  | Q5T5T6 |
|  | BAD96681 |
|  | NW\_000174 |
|  | 123796817 |
|  | 123797112 |
|  | 13277840 |
|  | 26332439 |
|  | 26343105 |
|  | 2736258 |
|  | 532621 |
|  | 731003 |
|  | 7329183 |
|  | 74196059 |
|  | 74198042 |
|  | 74198050 |
|  | 74212531 |
|  | 81914083 |
|  | 81914198 |
|  | AI325259 |
|  | 119608705 |
|  | 119608706 |
|  | 119608707 |
|  | 123996177 |
|  | 16549281 |
|  | 21619570 |
|  | 21708064 |
|  | 23503103 |
|  | 28175372 |
|  | 31874596 |
|  | 40352702 |
|  | 47419893 |
|  | 55958762 |
|  | 55958763 |
|  | 55958764 |
|  | 55958765 |
|  | 55958766 |
|  | 55959977 |
|  | 55959978 |
|  | 55959979 |
|  | 55959980 |
|  | 74740481 |
|  | 74744488 |
|  | 74744489 |
|  | 74745125 |
|  | 74756237 |
|  | 74756238 |
|  | 74759119 |
|  | 119608708 |
|  | 119608709 |
|  | 119608710 |
|  | 119608711 |
|  | 123993881 |
|  | 123227828 |
|  | 123227829 |
|  | 148676310 |
|  | 149039348 |

---

|  |  |
| --- | --- |
| Swiss-Prot ID | TRAF2\_HUMAN |
|  | TRAF2\_MOUSE |
|  | Q8CAQ7\_MOUSE |
|  | B5DFH7\_RAT |
|  | Q8C6X9\_MOUSE |
|  | Q3TWR1\_MOUSE |
|  | Q3U8L1\_MOUSE |
|  | A8K107\_HUMAN |

---

|  |  |
| --- | --- |
| Cell Localization | Cytoplasm |

---

|  |  |
| --- | --- |
| Ensembl ID | ENSG00000127191 |
|  | ENSP00000247668.2 |
|  | ENST00000247668.7 |
|  | ENSMUSG00000026942 |
|  | ENSMUSP00000109872.1 |
|  | ENSMUST00000114234.1 |
|  | ENSMUSP00000028311.6 |
|  | ENSMUST00000028311.12 |
|  | ENSRNOG00000006238 |
|  | ENSRNOP00000008253.6 |
|  | ENSRNOT00000008253.6 |
|  | ENSP00000247668 |
|  | ENST00000247668 |
|  | ENSMUSP00000109872 |
|  | ENSMUST00000114234 |
|  | ENSMUSP00000028311 |
|  | ENSMUST00000028311 |
|  | ENSRNOP00000008253 |
|  | ENSRNOT00000008253 |

---

|  |  |
| --- | --- |
| MGI ID | MGI:101835 |
|  | 101835 |
|  | 2138907 |

---

|  |  |
| --- | --- |
| RGD ID | 1310457 |

---

|  |  |
| --- | --- |
| Unigene ID | Hs.522506 |
|  | Mm.3399 |
|  | Rn.105232 |

---

|  |  |
| --- | --- |
| Homologene ID | 22520 |

---

|  |  |
| --- | --- |
| IPI ID | IPI00030278 |
|  | IPI00292635 |
|  | IPI00970911 |
|  | IPI00970988 |
|  | IPI00135901 |
|  | IPI00271450 |
|  | IPI00371898 |
|  | IPI00647767 |
|  | IPI00384922 |
|  | IPI00646169 |
|  | IPI00644377 |
|  | IPI00776867 |

---

|  |  |
| --- | --- |
| Homo sapiens Chromosome position | 9q34 |

---

|  |  |
| --- | --- |
| Ensembl Transcript ID | ENST00000247668 |

---

|  |  |
| --- | --- |
| ENSEMBL | ENSG00000127191 |

---

|  |  |
| --- | --- |
| FunctionalClass | protein binding |
|  | signal transducer |
|  | metal ion binding |
|  | receptor |
|  | zinc ion binding |
|  | protein complex binding |

---

|  |  |
| --- | --- |
| Mus musculus Chromosome position | 2 A3 |
|  | 2 |

---

|  |  |
| --- | --- |
| ProteinAtlas SubcellularLocation | Main location: Cytoplasm; Other location: ; Expression type: APE; Reliability: High |

---

|  |  |
| --- | --- |
| Rattus norvegicus Chromosome position | 3p13 |

---

|  |  |
| --- | --- |
| Ensembl Gene ID | ENSG00000127191 |

---

|  |  |
| --- | --- |
| ProteinAtlas NormalTissue | Tissue: kidney; Cell type: cells in tubules; Level: Low; Expression type: APE; Reliability: Low |
|  | Tissue: pancreas; Cell type: islets of Langerhans; Level: Low; Expression type: APE; Reliability: Low |
|  | Tissue: colon; Cell type: glandular cells; Level: Medium; Expression type: APE; Reliability: Low |
|  | Tissue: pancreas; Cell type: exocrine glandular cells; Level: Medium; Expression type: APE; Reliability: Low |
|  | Tissue: oral mucosa; Cell type: squamous epithelial cells; Level: Medium; Expression type: APE; Reliability: Low |
|  | Tissue: liver; Cell type: hepatocytes; Level: Low; Expression type: APE; Reliability: Low |
|  | Tissue: lung; Cell type: pneumocytes; Level: Low; Expression type: APE; Reliability: Low |
|  | Tissue: adrenal gland; Cell type: glandular cells; Level: Medium; Expression type: APE; Reliability: Low |
|  | Tissue: appendix; Cell type: glandular cells; Level: Medium; Expression type: APE; Reliability: Low |
|  | Tissue: appendix; Cell type: lymphoid tissue; Level: Low; Expression type: APE; Reliability: Low |
|  | Tissue: bone marrow; Cell type: hematopoietic cells; Level: Low; Expression type: APE; Reliability: Low |
|  | Tissue: breast; Cell type: glandular cells; Level: Medium; Expression type: APE; Reliability: Low |
|  | Tissue: bronchus; Cell type: respiratory epithelial cells; Level: Low; Expression type: APE; Reliability: Low |
|  | Tissue: cerebellum; Cell type: cells in granular layer; Level: Low; Expression type: APE; Reliability: Low |
|  | Tissue: cerebellum; Cell type: cells in molecular layer; Level: Low; Expression type: APE; Reliability: Low |
|  | Tissue: cerebellum; Cell type: Purkinje cells; Level: Low; Expression type: APE; Reliability: Low |
|  | Tissue: cerebral cortex; Cell type: glial cells; Level: Low; Expression type: APE; Reliability: Low |
|  | Tissue: cerebral cortex; Cell type: neuronal cells; Level: Low; Expression type: APE; Reliability: Low |
|  | Tissue: cervix, uterine; Cell type: glandular cells; Level: Medium; Expression type: APE; Reliability: Low |
|  | Tissue: cervix, uterine; Cell type: squamous epithelial cells; Level: None; Expression type: APE; Reliability: Low |
|  | Tissue: duodenum; Cell type: glandular cells; Level: Medium; Expression type: APE; Reliability: Low |
|  | Tissue: epididymis; Cell type: glandular cells; Level: Low; Expression type: APE; Reliability: Low |
|  | Tissue: esophagus; Cell type: squamous epithelial cells; Level: Medium; Expression type: APE; Reliability: Low |
|  | Tissue: fallopian tube; Cell type: glandular cells; Level: Medium; Expression type: APE; Reliability: Low |
|  | Tissue: gall bladder; Cell type: glandular cells; Level: Medium; Expression type: APE; Reliability: Low |
|  | Tissue: heart muscle; Cell type: myocytes; Level: Medium; Expression type: APE; Reliability: Low |
|  | Tissue: hippocampus; Cell type: glial cells; Level: Low; Expression type: APE; Reliability: Low |
|  | Tissue: hippocampus; Cell type: neuronal cells; Level: Low; Expression type: APE; Reliability: Low |
|  | Tissue: kidney; Cell type: cells in glomeruli; Level: Low; Expression type: APE; Reliability: Low |
|  | Tissue: lateral ventricle; Cell type: glial cells; Level: Low; Expression type: APE; Reliability: Low |
|  | Tissue: lateral ventricle; Cell type: neuronal cells; Level: Low; Expression type: APE; Reliability: Low |
|  | Tissue: liver; Cell type: bile duct cells; Level: Low; Expression type: APE; Reliability: Low |
|  | Tissue: lung; Cell type: macrophages; Level: Medium; Expression type: APE; Reliability: Low |
|  | Tissue: lymph node; Cell type: germinal center cells; Level: Medium; Expression type: APE; Reliability: Low |
|  | Tissue: lymph node; Cell type: non-germinal center cells; Level: Medium; Expression type: APE; Reliability: Low |
|  | Tissue: nasopharynx; Cell type: respiratory epithelial cells; Level: Medium; Expression type: APE; Reliability: Low |
|  | Tissue: ovary; Cell type: follicle cells; Level: Medium; Expression type: APE; Reliability: Low |
|  | Tissue: ovary; Cell type: ovarian stroma cells; Level: Medium; Expression type: APE; Reliability: Low |
|  | Tissue: parathyroid gland; Cell type: glandular cells; Level: Medium; Expression type: APE; Reliability: Low |
|  | Tissue: placenta; Cell type: decidual cells; Level: Medium; Expression type: APE; Reliability: Low |
|  | Tissue: placenta; Cell type: trophoblastic cells; Level: Medium; Expression type: APE; Reliability: Low |
|  | Tissue: prostate; Cell type: glandular cells; Level: Low; Expression type: APE; Reliability: Low |
|  | Tissue: rectum; Cell type: glandular cells; Level: Medium; Expression type: APE; Reliability: Low |
|  | Tissue: salivary gland; Cell type: glandular cells; Level: Medium; Expression type: APE; Reliability: Low |
|  | Tissue: seminal vesicle; Cell type: glandular cells; Level: Low; Expression type: APE; Reliability: Low |
|  | Tissue: skeletal muscle; Cell type: myocytes; Level: Medium; Expression type: APE; Reliability: Low |
|  | Tissue: skin; Cell type: epidermal cells; Level: Medium; Expression type: APE; Reliability: Low |
|  | Tissue: small intestine; Cell type: glandular cells; Level: Medium; Expression type: APE; Reliability: Low |
|  | Tissue: smooth muscle; Cell type: smooth muscle cells; Level: Low; Expression type: APE; Reliability: Low |
|  | Tissue: spleen; Cell type: cells in red pulp; Level: Low; Expression type: APE; Reliability: Low |
|  | Tissue: spleen; Cell type: cells in white pulp; Level: Medium; Expression type: APE; Reliability: Low |
|  | Tissue: stomach, lower; Cell type: glandular cells; Level: Medium; Expression type: APE; Reliability: Low |
|  | Tissue: stomach, upper; Cell type: glandular cells; Level: Medium; Expression type: APE; Reliability: Low |
|  | Tissue: testis; Cell type: cells in seminiferus ducts; Level: Medium; Expression type: APE; Reliability: Low |
|  | Tissue: testis; Cell type: Leydig cells; Level: Medium; Expression type: APE; Reliability: Low |
|  | Tissue: thyroid gland; Cell type: glandular cells; Level: Low; Expression type: APE; Reliability: Low |
|  | Tissue: tonsil; Cell type: germinal center cells; Level: Low; Expression type: APE; Reliability: Low |
|  | Tissue: tonsil; Cell type: non-germinal center cells; Level: Medium; Expression type: APE; Reliability: Low |
|  | Tissue: tonsil; Cell type: squamous epithelial cells; Level: Low; Expression type: APE; Reliability: Low |
|  | Tissue: urinary bladder; Cell type: urothelial cells; Level: Medium; Expression type: APE; Reliability: Low |
|  | Tissue: uterus, post-menopause; Cell type: cells in endometrial stroma; Level: Low; Expression type: APE; Reliability: Low |
|  | Tissue: uterus, post-menopause; Cell type: glandular cells; Level: Medium; Expression type: APE; Reliability: Low |
|  | Tissue: uterus, pre-menopause; Cell type: cells in endometrial stroma; Level: Low; Expression type: APE; Reliability: Low |
|  | Tissue: uterus, pre-menopause; Cell type: glandular cells; Level: Medium; Expression type: APE; Reliability: Low |
|  | Tissue: vagina; Cell type: squamous epithelial cells; Level: Medium; Expression type: APE; Reliability: Low |
|  | Tissue: vulva/anal skin; Cell type: epidermal cells; Level: Medium; Expression type: APE; Reliability: Low |

---
